# Large gap between attitude and action in tuberculosis preventive treatment among tuberculosis-related healthcare workers in eastern China

**DOI:** 10.3389/fcimb.2022.991400

**Published:** 2022-10-07

**Authors:** Fei Wang, Yanli Ren, Kui Liu, Ying Peng, Xinyi Chen, Bin Chen, Jianmin Jiang

**Affiliations:** ^1^ Tuberculosis Control Department, Zhejiang Provincial Center for Disease Control and Prevention, Hangzhou, China; ^2^ School of Public Health, Hangzhou Normal University, Hangzhou, China

**Keywords:** latent tuberculosis infection, healthcare workers, preventive treatment, attitudes, behaviors

## Abstract

Healthcare workers (HCWs) are at a high risk for latent tuberculosis infection (LTBI) because of occupational exposure, and the attitudes and behaviors of frontline tuberculosis (TB)-related HCWs toward preventive treatment of LTBI in eastern China remain unknown. This study aimed to explore the attitudes and actual behaviors of TB-related HCWs toward TB preventive treatment (TPT) and to analyze the relevant factors influencing the attitudes of HCWs. A stratified random sample of 28 TB-designated hospitals was selected in Zhejiang Province, China. All TB-related HCWs in the selected hospitals were recruited to answer questionnaires and were tested for LTBI by the TB interferon gamma release assay. TPT use was assessed two years after the survey. Univariate analysis and binary logistic regression models were used to analyze the factors influencing the TPT intention of HCWs. A total of 318 TB-related HCWs were recruited from 28 TB-designated hospitals; 62.3% of them showed positive attitudes toward TPT, while the rest were reluctant to treat positive LTBI prophylactically. binary logistic regression analysis revealed that the factors influencing the attitudes of HCWs were mainly education level, household income, history of alcohol consumption, and workplace. The IGRA test found that 35.2% (112/318) of HCWs tested positive for LTBI. Most people refused treatment because of drug side effects, followed by the belief that treatment was ineffective, wanting to wait until the onset of the disease, and that it was too much trouble to take the medication. According to the results of a follow-up survey, only one of these HCWs underwent TPT, and the consistency rate of attitudes and behaviors was 36.6% (41/112). This study reveals different attitudes toward TPT among TB-associated HCWs in eastern China and a large gap between attitudes and actual action. The management of HCWs with LTBI still needs further strengthening.

## Introduction

Latent tuberculosis infection (LTBI) is characterized by the presence of immune responses to previously acquired *Mycobacterium tuberculosis* infection without clinical evidence of active tuberculosis (TB) (Getahun et al., 2015). Approximately one-third of the global population has LTBI and 5–10% of them will develop active tuberculosis at some point in their lifetime without imposing any measures. Therefore, LTBI also represents a significant global burden for the strategic goal of TB eradication by 2050 (Houben and Dodd, 2016; Cohen et al., 2019).

TB healthcare workers (HCWs) are one of the populations most likely to develop LTBI, as they are those with frequent direct or indirect exposure to *M. tuberculosis*. Multiple studies have shown a significant correlation between TB infection and occupational exposure (Zhang et al., 2013; Shi et al., 2018; Graves et al., 2019). The prevalence of TB among nurses working in TB-related departments is 5.1 times higher than in the general population, and the incidence of LTBI among HCWs is 17–36% (Jo et al., 2008; Mok, 2016).

Tuberculosis preventive treatment (TPT) is a prophylactic treatment given to people who test positive for LTBI but have not yet progressed to active TB in order to lower their risk of developing active TB (WHO, 2020; Fox et al., 2021). TPT is considered a key intervention to reduce the prevalence of TB and has been prioritized by the World Health Organization in the “End TB Strategy.” However, most existing TPT regimens require continued dosing for 6–9 months, with the potential for a range of side effects, such drug-induced hepatitis (Smith et al., 2011; Ayelign et al., 2019). Therefore, acceptance rates for TPT are not high in patients with LTBI. Even though HCWs showed positive attitudes toward TPT and were willing to recommend it to their patients, their actual acceptance rate was lower than that of non-HCWs (Camins et al., 1996; Bhanot et al., 2012).

As one of the developed provinces in eastern China, Zhejiang Province has a 33.9% prevalence of LTBI among TB-related medical personnel (Chen et al., 2019). However, the attitudes and actual implementation of TPT by these positive LTBI medical personnel are unknown to us. How to reduce the prevalence of LTBI has become the next step in the TB prevention and control plan in China (WHO, 2018; WHO, 2020), starting with TPT intervention in populations that have close contact with *M. tuberculosis*. The aims of this study were to determine the attitudes and actual implementation of TPT among TB-related HCWs in Zhejiang Province and to provide evidence-based recommendations for preventive treatment of LTBI in China.

## Materials and methods

### Study design and participants

Zhejiang Province has an area of 105,500 km^2^ and 11 prefectures according to the administrative system of China. There are designated TB diagnosis and treatment hospitals in each prefecture and county in Zhejiang, including 13 at the prefecture level and 76 at the county level. This study was conducted using a stratified random sampling principle to select three medical institutions in each prefecture, including one prefectural-level hospital and two county-level hospitals. A total of 33 TB-designated hospitals were sampled, of which 5 hospitals refused to participate in the survey; therefore, the final number of hospitals included in the survey was 28. All TB-related HCWs in each hospital were invited to participate in the study. The inclusion criteria were as follows: HCWs who worked in the independent TB departments or shared sections that had direct contact with TB patients (e.g., X-ray rooms, pharmacies, and fee-charging rooms).

### Data collection

A questionnaire survey was conducted from July 2017 to July 2018 among 318 TB-related health care workers in 28 TB-designated hospitals. The questionnaire was designed based on the recommendations of the National TB Infection Control Manual and was developed in Chinese (Wang et al., 2010). The study was conducted by uniformly trained investigators from the provincial and prefectural Centers for Disease Control and Prevention, including epidemiologists, laboratory technicians, and infection control practitioners. The questionnaires were pre-tested and adjusted at a prefectural-level TB-designated hospital. The questions in the questionnaire were used to obtain demographic information (e.g., sex, age, educational attainment, marital status, etc.); individual characteristics, such as weight, height, Bacille Calmette–Guérin (BCG) vaccination status, and health-related behaviors; job characteristics and work experience; and questions related to TPT.

Interferon-gamma release assays (IGRA) were performed on HCWs immediately after the questionnaire and they were informed of the test results the next day. TB-IGRA was performed using a diagnostic kit for T cells infected with *M. tuberculosis* from Beijing Wantai Biopharmaceutical Co. (Beijing, China). After taking whole blood from the subject, the level of γ-interferon release in the sample was quantified by enzyme-linked immunosorbent assay to determine the infection status of the subject, and a test value of less than 14 was considered negative. This test reagent is widely used in China and was also used in our previous research (Chen et al., 2019).

Two years after completion of the questionnaire and IGRA testing, participating HCWs were followed up by telephone. The content of the return interview focused on whether the HCWs who tested positive for LTBI had carried out TPT.

### Statistical analysis

The data from the valid questionnaires were double-entered and checked with EpiData software (EpiData 3.01 for Windows; EpiData Association, Odense, Denmark). SPSS Statistics version 20 (IBM, Armonk, NY) was used to perform the statistical analysis. Univariate analysis was used to analyze the association between personal characteristics and TPT intentions. binary logistic regression analysis was used to analyze the factors affecting TPT.

### Ethics statement

The survey was approved by the Ethics Review Board of Zhejiang Provincial CDC. Signed informed consent forms were obtained from the respondents and personal information was kept confidential. All respondents were informed of the results of their LTBI tests, and a consultation was provided for those who needed further checks and TPT.

## Results

### Basic characteristics of TB-related HCWs

A total of 318 TB-related HCWs were included in the survey, of whom 95 (29.9%) were male and 223 (70.1%) were female (**Table 1**), with a mean age of 39.14 ± 8.97 years. Of these HCWs, 295 (92.8%) had college or higher education, 269 (84.6%) were married, 111 (34.9%) had an annual household income less than or equal to $15,000 and 64 (20.1%) of more than $30,000, 31 (9.7%) had a history of smoking, and 103 (32.4%) had a history of drinking. There were 221 (69.5%) HCWs with BMI in the normal range. Of those who participated in the survey, 179 (56.3%) HCWs had engaged in TB control for 9 years or fewer, and the largest number of HCWs (42.5%) were in the inpatient sector. There were 215 (67.6%) HCWs with previous BCG vaccination and 9 had TB prior to the survey but were currently cured.

**Table 1:** Basic characteristics of TB-related health care workers in Zhejiang Province.

**Variables**		**N**	**%**
Sex	Male	95	29.9
	Female	223	70.1
Age	<30	47	14.8
	30-39	130	40.9
	40-49	99	31.1
	≥50	42	13.2
Education level	Junior high school	7	2.2
	High school and secondary school	16	5
	College and above	295	92.8
Marital status	Married	269	84.6
	Unmarried	38	11.9
	Divorced or widowed	11	3.5
Annual household income (USD)	≤15000	111	34.9
	15000-30000	143	45
	>30000	64	20.1
Smoking history	No	287	90.3
	Yes	31	9.7
Drinking history	No	215	67.6
	Yes	103	32.4
BMI	<18.5	20	6.3
	18.5-24	221	69.5
	≥24	77	24.2
Years of TB-related work	≤9 years	179	56.3
	9-19 years	102	32.1
	19-28 years	32	10.1
	>28 years	5	1.6
Position	Junior position	103	32.4
	Intermediate position	132	41.5
	Senior position	83	26.1
Work place	Outpatient department	49	15.4
	Inpatient department	135	42.5
	TB laboratory	40	12.6
	Other	94	29.6
BCG vaccination	No	103	32.4
	Yes	215	67.6
TB history	No	309	97.2
	Yes	9	2.8

### Analysis of different characteristics of TB-related HCWs attitudes toward TPT

After surveying TB-related HCWs about their willingness to take TPT, 198 (62.3%) were willing to take TPT (63 males and 135 females, **Table 2**). There were also 120 (37.7%) who did not want to take TPT (32 males and 88 females). However, there was no sex bias in willingness to take TPT (χ2 = 0.946, P = 0.331). There was a significant difference in TB-related HCWs’ attitudes toward willingness to take TPT when assessed by education level (χ2 = 7.374, P = 0.025), with less-educated HCWs being more reluctant to take TPT. In addition, annual household income and drinking history were significantly associated with willingness to take TPT (χ2 = 9.518, P = 0.009; χ2 = 10.12, P = 0.001; respectively), and those with higher annual household income were less inclined to take TPT. None of the other characteristics were significantly associated with the attitudes of HCWs regarding their willingness to take TPT.

**Table 2:** Different characteristics of TB-related health workers willingness to take prophylaxis medication.

Variables		prophylaxis take medicine				χ2	P	OR
		willing	%	unwilling	%			
Sex	Male	63	66.3	32	33.7	0.946	0.331	0.779
	Female	135	60.5	88	39.5			
Age	<30	32	68.1	15	31.9	2.763	0.43	0.956
	30-39	76	58.5	54	41.5			
	40-49	66	66.7	33	33.3			
	≥50	24	57.1	18	42.9			
Education level	Junior high school	1	14.3	6	85.7	7.374	0.025	2.182
	High school and secondary school	9	56.3	7	43.8			
	College and above	188	63.7	107	36.3			
Marital status	Married	169	62.8	100	37.3	0.354	0.838	0.922
	Unmarried	22	57.9	16	42.1			
	Divorced or widowed	7	63.6	4	36.4			
Annual household income (USD)	≤15000	59	53.2	52	46.8	9.518	0.009	1.654
	15000-30000	90	62.9	53	37.1			
	>30000	49	76.6	15	23.4			
Smoking history	No	179	62.4	108	37.6	0.014	0.906	0.955
	Yes	19	61.3	12	38.7			
Drinking history	No	121	56.3	94	43.7	10.12	0.001	2.301
	Yes	77	74.8	26	25.2			
BMI	<18.5	12	60	8	40	1.203	0.548	1.249
	18.5-24	134	60.6	87	39.4			
	≥24	52	67.5	25	32.5			
Years of TB-related work	≤9 years	110	61.5	69	38.5	0.225	0.973	1.059
	9-19 years	64	62.7	38	37.3			
	19-28 years	21	65.6	11	34.4			
	>28 years	2	40	3	60			
Position	Junior position	64	62.1	39	37.9	0.136	0.934	1.034
	Intermediate position	81	61.4	51	38.6			
	Senior position	53	63.9	30	36.1			
Work place	Outpatient department	34	69.4	15	30.6	4.819	0.186	1.006
	Inpatient department	81	60	54	40			
	TB laboratory	20	50	20	50			
	Other	63	67	31	33			
BCG vaccination	No	58	56.3	45	43.7	2.298	0.13	1.448
	Yes	140	65.1	75	34.9			
TB history	No	194	62.8	115	37.2	0.593	0.441	0.474
	Yes	4	44.4	5	55.6			

### Analysis of factors affecting TB-related HCWs willingness to take TPT

Binary logistic regression was used to analyze the influential factors affecting TB-related HCWs’ attitudes toward taking TPT. Sex and age had no effect on HCWs’ attitudes toward TPT (**Table 3**). HCWs with college or higher education were more likely to receive TPT than HCWs with junior high school education (odds ratio [OR] = 17.261, 95% confidence interval [CI] = 1.58–188.527, P = 0.02), and there was an effect on HCWs’ attitudes toward TPT when annual household income exceeded $30,000 (OR = 2.297, 95% CI = 1.05–5.026, P = 0.037). HCWs with a history of alcohol consumption were more willing to take TPT compared to HCWs who had never drank (OR = 2.448, 95% CI = 1.323–4.529, P = 0.004). However, smoking history had no effect on the attitudes of HCWs. In addition, HCWs working in TB laboratories were more reluctant to take TPT than those working in TB outpatient departments (OR = 0.336, 95% CI = 0.124–0.91, P = 0.032). No other factors had an effect on the attitude of TB-related HCWs towards TPT.

**Table 3:** Multivariate regression analysis of the influencing factors of TB-related health workers willingness to take prophylaxis medicine.

Variables		OR	95%CI	P
Sex	Male	1		
	Female	0.761	0.401~1.445	0.404
Age	<30	1		
	30-39	0.58	0.222~1.518	0.267
	40-49	0.883	0.288~2.708	0.828
	≥50	0.805	0.207~3.137	0.755
Education level	Junior high school	1		
	High school and secondary school	12.193	0.973~152.826	0.053
	College and above	17.261	1.58~188.527	0.02
Marital status	Married	1		
	Unmarried	0.786	0.317~1.95	0.604
	Divorced or widowed	1.378	0.307~6.198	0.676
Annual household income (USD)	≤15000	1		
	15000-30000	1.629	0.916~2.897	0.097
	>30000	2.297	1.05~5.026	0.037
Smoking history	No	1		
	Yes	0.428	0.157~1.169	0.098
Drinking history	No	1		
	Yes	2.448	1.323~4.529	0.004
BMI	<18.5	1		
	18.5-24	0.863	0.297~2.513	0.787
	≥24	1.056	0.324~3.441	0.927
Years of TB-related work	≤9 years	1		
	9-19 years	0.842	0.458~1.547	0.579
	19-28 years	1.216	0.452~3.272	0.699
	>28 years	0.897	0.104~7.749	0.921
Position	Junior position	1		
	Intermediate position	0.746	0.367~1.518	0.419
	Senior position	0.478	0.194~1.179	0.109
Work place	Outpatient department	1		
	Inpatient department	0.625	0.285~1.368	0.24
	TB laboratory	0.336	0.124~0.91	0.032
	Other	0.75	0.329~1.709	0.493
BCG vaccination	No	1		
	Yes	1.206	0.703~2.068	0.497
TB history	No	1		
	Yes	0.34	0.075~1.543	0.162

### Consistency analysis of TB-related HCWs’ attitudes toward taking TPT and reasons for reluctance to take TPT

The IGRA test administered at the same time as this survey found that 112 (35.2%) of HCWs tested positive for LTBI and 206 (64.8%) tested negative. All HCWs with LTBI were further checked to exclude cases of active TB. Of the 112 HCWs who tested positive by IGRA, 72 indicated a willingness to take TPT at the time of the survey. However, after a follow-up survey two years later, only one HCW took TPT, resulting in a consistency rate of attitudes and behaviors of 36.6% (41/112). In the follow-up survey of reasons for HCWs who did not take TPT, only 48 HCWs were willing to answer (**Table 4**). Of these, 52.1% of HCWs felt that TPT was effective but prone to adverse effects and therefore was not necessary, 16.7% believed that TPT was ineffective, 4.1% were reluctant to act because it was too much trouble to take the medication, and 14.6% thought it would be better to wait until the onset of TB before taking the medication.

**Table 4:** Analysis of the reasons why health care workers think preventive medicine is unnecessary.

Reasons	N		%
Perceived ineffectiveness of preventive medication	8		16.7
Considered effective but prone to adverse reactions	25		52.1
Considered effective but troublesome to take	2		4.1
It is better to wait until the onset of the disease before taking medication	7		14.6
Others	6		12.5
Total	48		100

## Discussion

TB is one of the most common occupational risks for HCWs, who are more likely to be exposed to TB patients and are at increased risk of developing TB. This study showed that 62.3% (198/318) of TB-related HCWs were willing to TPT, but only 1 LTBI-positive HCW implemented prophylactic medication in the following two years. The survey revealed that most HCWs expressed a strong willingness to accept TPT for LTBI. However, the reality is that HCWs are inconsistent in their attitudes and behaviors toward TPT. In addition, the prevalence of LTBI in HCWs in this study was 35.2% (112/318).

Our survey represents the status of TPT acceptance of TB-related HCWs in Zhejiang Province in eastern China. In studies in Germany and Korea (Zielinski et al., 2021; Min et al., 2022), one-third of HCWs with LTBI received and completed a course of TPT. This level of acceptance was much higher than in China, which may be related to the use of a short course of treatment (3 months) in both countries (Min et al., 2022). The lower TB burden in developed countries has led to a correspondingly greater focus on LTBI and important national strategies for LTBI, and governments provide more TPT measures and have higher accessibility of measures (Linas et al., 2011). Thus, high levels of detection and successful treatment have been achieved in countries with a low TB burden, thereby successfully hindering the possibility of LTBI developing into active TB (Chee et al., 2018). However, the focus in high-burden countries is on patients, and resources and measures for TPT of patients with LTBI are not sufficient (Pang, 2014). In an Australian study (Pathak et al., 2016), 13% of HCWs actually received TPT. In general, there are varying degrees of variation between the attitudes and behaviors of HCWs in each country regarding TPT for LTBI. They all have very positive attitudes, but their actual behaviors do not reflect this. Countries with high TB burden have a lower rate of actual completion of TPT (Swift et al., 2020).

The higher the education level of HCWs, the more willing they were to accept TPT, which is contrary to the findings of a study in the United States (Swift et al., 2020) indicating that doctors and scientists at the doctoral level had the lowest level of acceptance of TPT compared to HCWs at other educational levels. However, the attitudes of medical personnel with different levels of education towards TPT in this study are consistent with those of the general population (Colson et al., 2013). This reflects the need to enhance HCWs’ knowledge of LTBI and TPT. Alcohol consumption is also a factor that influences the different attitudes of HCWs towards TPT, with those who used to drink or still drink being more willing to take TPT, and we speculate that these HCWs are more concerned about the high risk of developing TB (Louvet and Mathurin, 2015; Lackner and Tiniakos, 2019). Therefore, they are more willing to take TPT if they can avoid developing active TB. This study also found that the higher the annual household income, the more willing HCWs are to take TPT. People with higher incomes are usually more invested in their health, and TPT avoids the greater risk associated with the development of TB. In addition, HCWs working in a TB laboratory were more unwilling to take TPT, and they may be less aware of the clinical consequences than other sectors that are in direct contact with patients. Despite their exposure to *M. tuberculosis*, HCWs working in TB laboratories had the lowest infection rates compared to TB clinics and wards (Chen et al., 2019).

This study also provides further analysis of the reasons why TPT was deemed unnecessary by HCWs. Most HCWs disagree with TPT because TB drugs have certain side effects that can cause some degree of harm to the body, including the nervous system, liver, kidneys, ears, and blood (Borisov et al., 2019). One study showed that TPT with a short course of rifapentine/isoniazid or rifampin had fewer side effects and was more acceptable to HCWs than other treatment programs (Arguello Perez et al., 2017). Secondly, some people think that TPT is ineffective, and some people think that it is better to wait until the onset of disease. A small number of people refused to take TPT because they thought it was too troublesome. In addition, a study showed that the inability to guarantee the efficacy of TPT, the potential for drug resistance, and the lack of financial and policy support were also major barriers to the acceptance of TPT by healthcare professionals (Fa et al., 2022).

There are some limitations to this study. First, although the respondents of the survey had covered about one-third of the total TB-related HCWs in Zhejiang, the sample size included was somewhat small, and an expansion of the sample size would provide a clearer picture of whether the factors are truly statistically significant for the willingness of medical personnel to take TPT. Secondly, this study was conducted in Zhejiang Province, which only represents the current situation of HCWs in eastern China and cannot represent the national situation. Finally, as the world trends toward TB eradication and national policies continue to change, HCWs’ attitudes toward LTBI prophylaxis may change, and so the results of this study are representative of the period surveyed.

## Conclusion

This study identified the inconsistency between the attitudes and behaviors of HCWs in their approach to TPT, particularly in LTBI-positive HCWs who were willing to take TPT but did not actually take it. Regarding HCWs who test positive by IGRA, their knowledge of TB needs to be enhanced to prevent it from developing into active TB. The concept and knowledge of TPT still need to be further promoted among HCWs. Resources and financial support are needed to promote TPT for HCWs in the future.

## Data availability statement

The raw data supporting the conclusions of this article will be made available by the authors, without undue reservation.

## Ethics statement

The studies involving human participants were reviewed and approved by ethics review committee of Zhejiang Center for Disease Control and Prevention. The patients/participants provided their written informed consent to participate in this study.

## Author contributions

FW, conceptualization, methodology, writing - original draft. YR, software and writing- original draft. KL, visualization and investigation. YP, supervision. XC, software and validation. BC, writing- reviewing and editing, project administration. JJ, writing – review, editing, and supervision. All authors contributed to the article and approved the submitted version.

## Funding

This study was supported by the National Health and Health Commission Scientific Research Fund (WKJ-ZJ-2118).

## Acknowledgments

We would like to thank the staff from the local Centers for Disease Control and Prevention and local TB-designated hospitals for their help in the field survey.

## Conflict of interest

The authors declare that the research was conducted in the absence of any commercial or financial relationships that could be construed as a potential conflict of interest.

## Publisher’s note

All claims expressed in this article are solely those of the authors and do not necessarily represent those of their affiliated organizations, or those of the publisher, the editors and the reviewers. Any product that may be evaluated in this article, or claim that may be made by its manufacturer, is not guaranteed or endorsed by the publisher.
